# A rare case of accessory mitral valve tissue causing left ventricular outflow tract obstruction associated with parachute mitral valve, ventricular septal defect, bicuspid aortic valve, unruptured aneurysm of aortic sinus: a case report

**DOI:** 10.1093/ehjcr/yty082

**Published:** 2018-07-16

**Authors:** Yanan Li, Yanbin Hu, Jiaxiang Wang, Lin Liu

**Affiliations:** 1 Department of Cardiovascular Ultrasound, Henan Provincial People’s Hospital, People’s Hospital of Zhengzhou University, Zhengzhou, China; 2 Department of Cardiac Surgery, Henan Provincial People’s Hospital, People’s Hospital of Zhengzhou University, Zhengzhou, China

**Keywords:** Accessory mitral valve tissue, Left ventricular outflow tract obstruction, Parachute mitral valve, Case report

## Abstract

**Background:**

Accessory mitral valve tissue rarely causes left ventricular outflow tract obstruction in adults. It is often associated with other cardiac and vascular congenital malformations. Here, we report the rarest presentation of accessory mitral valve tissue (AMVT) causing left ventricular outflow tract obstruction.

**Case summary:**

A 22-year-old female patient presented with a history of shortness of breath and chest pain for more than 5 years. A diagnosis of AMVT with parachute mitral valve, ventricular septal defect (VSD), bicuspid aortic valve, unruptured aneurysm of aortic sinus, and left ventricular outflow tract obstruction was made. Successful closure of VSD with mitral valve replacement, excision of AMVT, and repair of the aortic sinus were performed. The post-operative course was uneventful, and an echocardiogram showed complete resection of the accessory mitral valve, no residual shunt and no left ventricular outflow gradient. Additionally, the peak gradient of rapid filling phase and atrial systolic phase across the prosthetic mitral valve were 16 mmHg and 4 mmHg, respectively. The peak velocity across left ventricular outflow tract was 1.4 m/s.

**Discussion:**

Accessory mitral valve tissue is associated with other cardiac abnormalities and is usually diagnosed in the first or second decade of life. It is responsible for left ventricular outflow tract obstruction. The obstruction can occur in the early period of life due to continued deposition of fibrous tissues within left ventricular outflow tract. Accessory mitral valve tissue should be considered a rare but important cause of left ventricular outflow tract obstruction.

**Learning points**

- Accessory mitral valve tissue is a rare congenital anomaly of endocardial cushion development. It rarely causes left ventricular outflow tract obstruction in adults and is often associated with other cardiac and vascular congenital malformations.
- Accessory mitral valve tissue has been described as parachute-like, sail-shaped, sac-like, leaflet-like, or as a sheet.

## Introduction

Accessory mitral valve tissue (AMVT) is a rare congenital anomaly that develops because of an incomplete separation of the mitral valve from the endocardial cushion tissue.^1^ AMVT is responsible for left ventricular outflow tract (LVOT) obstruction and is associated with other cardiac and vascular congenital malformations.^2^ We describe the case of a 22-year-old female patient diagnosed with parachute mitral valve (PMV), ventricular septal defect (VSD), bicuspid aortic valve (BAV), unruptured aneurysm of aortic sinus and LVOT obstruction produced by an AMVT. This combination of symptoms has not been reported previously in an adult.

## Timeline

**Table:** 

24 October 2017	The patient was referred to the Department of Cardiology
26 October 2017	Transthoracic echocardiography was performed
14 November 2017	Surgical treatment was carried out. (The operation time was 09:40–15:10.) A pre-operative and post-operative transoesophageal echocardiography was performed to evaluate surgical curative effects
22 November 2017	The first transthoracic echocardiography was performed after the operation
25 November 2017	The patient discharged from our hospital

## Case report

A 22-year-old female patient was referred to the Department of Cardiology with exertional shortness of breath and a history of congenital heart disease with mitral stenosis (MS), VSD and pulmonary hypertension as an outpatient. Five days previously, her condition had deteriorated rapidly, the patient became progressively dyspnoeic and developed orthopnoea, and she was unable to perform daily activities due to severe shortness of breath associated with exertional atypical chest pain. An electrocardiogram showed an ectopic rhythm (78 b.p.m./m), rapid atrial arrhythmias, and a left QRS axis. Upon physical examination, her blood pressure was found to be normal (100/60 mmHg) and the cardiac auscultation showed a 3/6 systolic murmur along the left sternal border. On auscultation of the chest, there were normal vesicular breath sounds. Scoliosis was observed in chest findings. There were no peripheral oedema and jugular venous distention. Blood and biochemical laboratory tests revealed leucocytosis, white blood cell count 10.18 × 10^9^ cells/L (normal value: 3.5–9.5 × 10^9^ cells/L), elevated C-reactive protein levels 25.36 mg/L (normal value: 1–10 mg/L) and NT-proBNP 628 ng/L (normal value: 133–450 ng/L). Chest radiography showed a cardiothoracic ratio of 60%, pulmonary congestion, bilateral pleural thickening and scoliosis.

**Figure 1:**
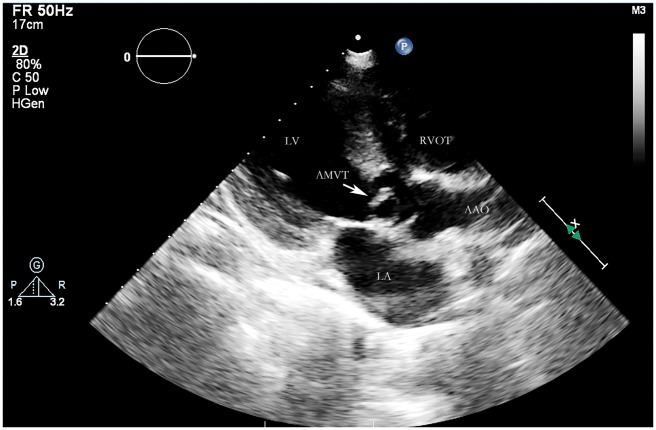
The parasternal long-axis view showed a fixed accessory mitral valve tissue attached to the anterior leaflet, which caused left ventricular outflow tract obstruction.

**Figure 2:**
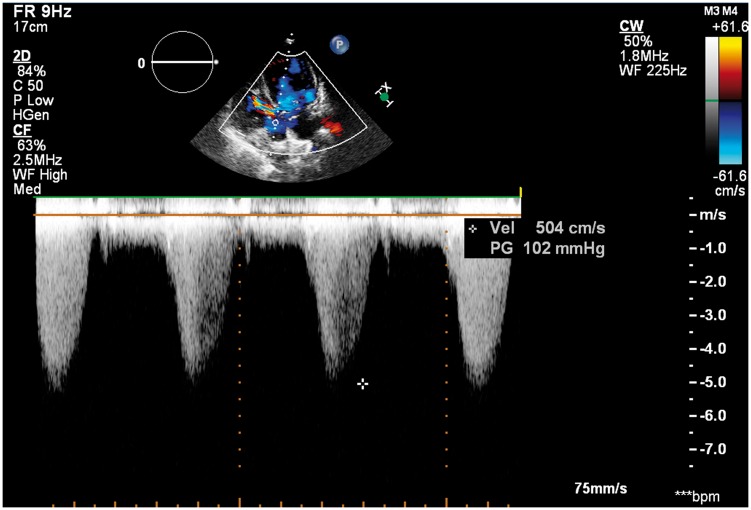
Apical five-chamber view: continuous wave Doppler examination showed the peak flow velocity across left ventricular outflow tract was 5.04 m/s.

Next, we performed a transthoracic echocardiography (TTE) that revealed an abnormal membranous structure (1.3 cm × 1.2 cm) attached to the ventricular side of anterior mitral valve (MV) leaflet, with a subaortic chordal attachment. The abnormal tissue was similar to a MV leaflet. The parasternal short-axis view showed the relationship between the normal MV and the accessory valve (*Figure 1*). Therefore, we diagnosed it as AMVT. The peak blood flow velocity across LVOT had accelerated to 5.0 m/s (*Figure 2*). The MV had a parachute-like appearance. There were two normally positioned papillary muscles in the left ventricle, but they did not have chordae tendineae inserted into it. The chordae tendineae were located in the left ventricular wall between the two papillary muscles (Supplementary material online, *Video S1*). Therefore, we described it as a parachute-like MV. Doppler assessment yielded a peak MV gradient of 27 mmHg and a mean MV gradient of 16 mmHg. Pressure half-time method yielded a valve area of 0.8 cm^2^. There was also a mild degree of mitral regurgitation. In addition, echocardiography revealed a peri-membranous VSD (Supplementary material online, *Videos S2* and *S3*) and BAV. The diameter of the VSD was 1.0 cm, and the shunt flow through VSD was left-to-right with a peak velocity of 4.5 m/s. From these findings, we hypothesized that LVOT obstruction was due to the accessory MV, MS was due to PMV and the left-to-right shunt was due to VSD. The pulmonary and tricuspid valves were normal. The pulmonary artery systolic pressure was slightly high (44 mmHg). There was also a mild dilatation of the left atrium (4.2 cm) and left ventricle (left ventricle end-diastolic diameter = 5.2 cm). Systolic function of the left ventricle was estimated to be within normal limits (ejection fraction = 60%). right ventricle function as well as the dimensions and morphology of the aortic arch and the ascending and descending aorta were normal.

In addition, we performed transoesophageal echocardiography (TOE) that confirmed the TTE findings, showing a fixed, membrane-like structure arising from the anterior mitral leaflet causing the LVOT obstruction (Supplementary material online, *Video S4*). This also confirmed the presence of PMV and VSD (Supplementary material online, *Videos S5* and *S6*). Transoesophageal echocardiography showed a BAV with only two sinuses: left coronary and right coronary. The right coronary sinus appeared aneurysmal, but not ruptured (Supplementary material online, *Video S7*). The valve area of BAV was 2.5 cm^2^ by planimetry, BAV was functioning normally with no aortic valve stenosis or regurgitation.

**Figure 3:**
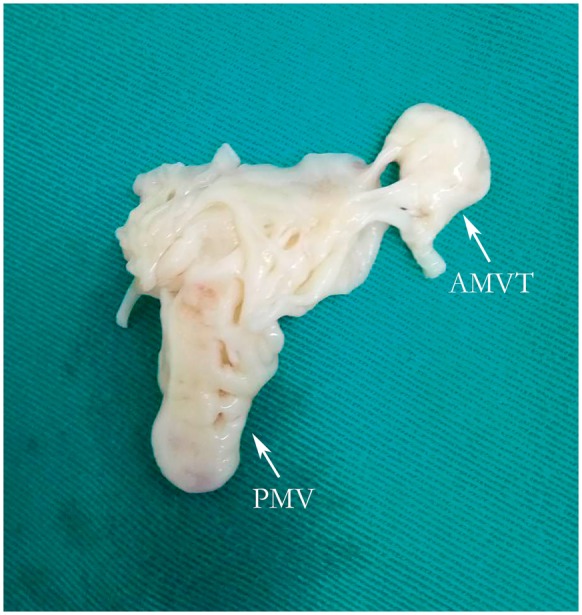
AMVT and PMV after resection. AMVT, accessory mitral valve tissue; PMV, parachute mitral valve.

After a comprehensive clinical evaluation, elective surgical treatment was performed because of the presence of VSD, severity of the MV stenosis and LVOT obstruction. The BAV was functioning normally and was not replaced. The operative findings correlated with the imaging diagnosis. During the operation, excision of the AMV and MV replacement were performed. In addition, the peri-membranous VSD and aneurysm of the aortic sinus were repaired. Grossly, the membranous structure specimens confirmed that it was AMVT (*Figure 3*). Histological examination showed valvular tissue associated with mucous degeneration and inflammatory cell infiltration.

A post-operative echocardiography showed complete resection of AMV, no residual shunt, no left ventricular outflow gradient or mitral regurgitation. The mosaic flow signals in the left ventricular outflow were diminished using colour Doppler, and the peak blood flow velocity decreased to 1.4 m/s. The peak pressure of rapid filling phase and atrial systolic phase across the prosthetic MV were 16 mmHg and 4 mmHg, respectively. The post-operative course was uneventful, and the patient was most recently reviewed 1 week, 1 month, 3 months after the surgical procedure by ultrasound, electrocardiogram, and chest radiography. The function of BAV was evaluated by ultrasound regularly. In the most recent follow-up, the patient’s shortness of breath disappeared, with no further episodes of chest pain.

In this study, we report an extremely rare case of AMVT causing LVOT obstruction associated with PMV, VSD, BAV, and an unruptured aneurysm of the aortic sinus. This combination of symptoms has not been previously reported in literature.

## Discussion

Accessory mitral valve tissue is a rare congenital anomaly of endocardial cushion development and is commonly associated with other congenital cardiac anomalies. Accessory mitral valve tissue arises from the anterior mitral leaflet and causes LVOT obstruction. Transthoracic echocardiography and TOE are considered the primary imaging modalities for AMVT diagnosis and patient follow-up.^3^ We performed TTE and TOE that revealed AMVT attached to the anterior mitral leaflet, with a subaortic chordal attachment, resulting in a severe increase in LVOT gradient (118 mmHg). Accessory mitral valve tissue has been described as parachute-like, sail-shaped, sac-like, leaflet-like, or as a sheet. Prifti *et al.*^4^ classified this abnormality into a fixed type (Type I) or mobile type (Type II) depending on the morphology of the abnormal tissue. Fixed and mobile types were further divided into the nodular type (Type IA) and the membranous type (Type IB) and into the pedunculated (Type IIA) type and the leaflet type (Type IIB), respectively. The leaflet type was subdivided into the rudimentary chordae tendineae type (Type IIB1) and the well-developed chordae tendineae type (Type IIB2). Our patient had a fixed membranous type leaflet-like structure directly attached to the endocardium with chordae tendineae; therefore, our case was classified as Type IB.

Accessory mitral valve tissue is associated with other cardiac abnormalities. Manganaro *et al.*^5^ identified 104 patients who were diagnosed with AMVT from 1963 to 2012. Most patients (86.6%) had signs of LVOT obstruction, and AMVT was frequently associated with VSD (19.2%), followed by subaortic membrane (9.6%), LV hypertrophy (8.6%), and transposition of the great arteries (7.7%). In this case study, we report the first and the rarest presentation of AMVT in a 22-year-old female patient diagnosed with PMV, VSD, BAV, an unruptured aneurysm of the aortic sinus, and LVOT obstruction produced by AMVT.

The obstruction can occur in the early period of life due to continued deposition of fibrous tissues within LVOT. Accessory mitral valve tissue is usually diagnosed in the first or second decade of life, and the main symptoms are exercise intolerance with dyspnoea, chest pain, and syncope.^6^ Patients with AMVT become symptomatic when the mean pressure gradient across LVOT reaches >50 mmHg.^7^ The peak LVOT pressure gradient of our patient was 100 mmHg, and LVOT stenosis was confirmed to be severe in the parasternal short-axis images. From a surgical point of view, early surgical removal is recommended because the pressure gauge gradient might increase with age.

## Conclusion

Accessory mitral valve tissue rarely causes LVOT obstruction in adults. It is often associated with other cardiac and vascular congenital malformations. It should be considered a rare but important cause of LVOT obstruction.

## Supplementary Material

Supplementary Video 1

Supplementary Video 2

Supplementary Video 3

Supplementary Video 4

Supplementary Video 5

Supplementary Video 6

Supplementary Video 7
